# Professor Michael Barnes, Honorary Professor of Neurological Rehabilitation, University of Newcastle, U.K.: Adapted Interview from the 12^th^ World Congress for NeuroRehabilitation (WCNR), Vienna, 2022

**DOI:** 10.25122/jml-2023-1005

**Published:** 2023-02

**Authors:** Stefana-Andrada Dobran, Alexandra Gherman

**Affiliations:** 1 RoNeuro Institute for Neurological Research and Diagnostic, Cluj-Napoca, Romania; 2 Sociology Department, Babes-Bolyai University, Cluj-Napoca, Romania

**Interviewer: Stefana-Andrada Dobran**

**Interviewee: Michael Barnes** °

° **Honorary Professor of Neurological Rehabilitation**, the University of Newcastle, Newcastle upon Tyne, United Kingdom

**°** Chair, **The Medical Cannabis Clinicians Society (MCCS)****, London, United Kingdom**

The MCCS is a not-for-profit society providing training, expert guidance, and peer support for the first clinicians prescribing life-changing medical cannabis treatments in the UK. It exists to equip clinicians with the education, evidence, expert support network, and prescription guidance they need to safely and confidently prescribe medical cannabis for patients.

° **Chair**, Clinical Governance, NRC Medical Experts, United Kingdom

With over 20 years of experience as a medico-legal expert, Professor Barnes has authored more than 3000 court reports. In addition, as a Managing Partner, he provides supervision and mentoring to members of the chambers. The NRC offers specialized medico-legal services that cover all aspects of neurorehabilitation. Its team of experienced rehabilitation experts provides assessment, court reports and acts as expert witnesses across a range of specialisms, including neurorehabilitation medicine, neuropsychiatry, neuropsychology, neurology, neuro-physiotherapy, neuro-occupational therapy and speech therapy and behavioral nursing, traumatic brain injury, and spinal injury.

° **Consultant** Neurologist and Consultant in Rehabilitation Medicine, NHS, United Kingdom

Professor Barnes has held various roles in the NHS, including serving as the Chief Executive and Medical Director of NHS organizations responsible for managing rehabilitation and community services. He established the Regional Neurorehabilitation Centre in Newcastle (Hunters Moor and, latterly, Walkergate Park).

**S.A.D.:** Dear Professor Barnes, we are here, in Vienna, for the 12^th^ World Congress for NeuroRehabilitation, organised by the World Federation for NeuroRehabilitation. What is your first-hand opinion of the event so far, and have you participated in any previous editions?

**M.B.:** It looks really comprehensive. It is nice to see so many parallel tracks; there are so many workshops, so many seminars, and plenary lectures; it is really comprehensive, the most comprehensive congress I’ve been to. I have been to them all, in fact. We set the first one in 1996, in the U.K., and I’ve been to every one since. This looks like the most comprehensive World Federation Congress. Great, well done to the organisers!

**S.A.D.:** What do you believe is the overarching theme of this year’s Congress?

**M.B.:** I’ve looked at the programme, and it doesn’t seem to have one theme. Some congresses have a theme, but this doesn’t. And I think that’s actually good because it’s very comprehensive, it goes through all aspects of neurorehabilitation, right across the spectrum: multidisciplinarity work, it has an emphasis on young neurorehabilitation – doctors and therapists, it has a lot of emphasis on the developing world. I think it is actually good not to have one specific theme which would exclude other themes; it is good to see a totally comprehensive programme.

**S.A.D.:** Thank you for the insight! From your perspective, what is the role of hybrid multidisciplinary events in developing neurorehabilitation research and practice, and which similar avenues are worth exploring?

**M.B.:** Hybrid events, of course, came out as a result of COVID. I think it is great that we are able to meet face-to-face to an extent. It would be nice to see these big international congresses always being hybrid from now on. There’s great value in people meeting together, sitting over coffee, and talking about other things which you can’t do over Zoom or Teams or whatever it is. But equally, if one would force people to come to a meeting like this, a lot of people won’t be able to, either because they can’t afford it, or they do not have the time, or travel would be an issue. I think there is a good role for hybrid conferences always, from now on. As many people as possible coming face-to-face, but let’s not exclude those who can’t come to meetings like this; let’s involve those through hybrid mechanisms. So, that’s a good thing [that] I think it will stay.

**S.A.D.:** Could you please describe the evidence base for phytocannabinoid treatments?

**M.B.:** As I moved on, to an extent, from neurorehabilitation broadly – I used to do just brain injury and multiple sclerosis and botulinum toxin, I am now really just involved in cannabis development, medical cannabis for neurorehabilitation and neurological conditions. So, the evidence is now really quite strong that cannabis generally helps. It helps pain – number 1, it helps anxiety – number 2, it helps epilepsy, particularly for some of the childhood drug-resistant epilepsies – number 3, and there are a lot of other conditions for which it does seem to help, but unfortunately, the evidence is not quite as robust as it is for pain or anxiety and epilepsy, and some other neurological conditions, like spasticity. There is a lot of real-world evidence, an awful lot; there are about 40.000 publications on the real-world evidence [regarding] the usefulness of medical cannabis. We are lacking in the classic pharmaceutical approach of double-blind placebo control studies, but cannabis doesn’t lend itself to that approach. So, we do need a different view on how to evaluate evidence for cannabis. And the evidence for cannabis for those conditions is pretty strong, so I think it has a really important role to play in the management of a lot of neurological conditions, and I’d like to see that more accepted. There is still a little bit of a stigma about cannabis in many countries and through many conditions – and neurologists are one of those who are perhaps more sceptical about it than others. I don’t think we need to be sceptical; it is a very useful medicine, a very safe medicine, and a medicine that can influence many long-term neurological conditions.

**S.A.D.:** Are patients and physicians aware of the potential use of medical cannabis?

**M.B.:** A quick answer is "no". Doctors and others – nurses and therapists are not really taught about cannabis when they are young doctors or young nurses. There needs to be more training and more knowledge. A lot of people speak out of ignorance because they do not know about cannabis, they view it as a recreational drug, and therefore it does not have medical value – that is not true. What we really do need to do, and I would like to see it [done] through the World Federation, is teaching and training, which is why I am doing a teaching session on Friday here, at the Congress, on cannabis. The doctors and multidisciplinary teams need to know much more about it so they can accept it or reject it, but at least speak from a position of knowledge rather than from a position of ignorance.

**S.A.D.:** On the same theme, do you think that clinical guidelines for medical cannabis are needed?

**M.B.:** Yes, very much so. It is totally different from normal medicine, is not like a new drug that has come out; we need good quality guidelines. We have some in some countries, in the U.K., we have guidelines; I’d like to see the World Federation produce global guidelines, sensible, balanced guidelines that tell doctors what the evidence is and what the evidence isn’t. We are not pushing cannabis as a cure-all medicine for everything, but it does have a place. I think the World Federation should produce guidelines that tell doctors, therapists, and nurses what their place is. I would love to see international, global guidelines.

**S.A.D.:** Thank you very much for the insight, and thank you very much for the interview!

**M.B.:** Thank you for asking me!

